# Serum creatinine to cystatin C ratio as monitoring biomarker in Chinese adult spinal muscular atrophy: a prospective cohort study

**DOI:** 10.1186/s13023-025-03730-3

**Published:** 2025-05-02

**Authors:** Sihui Chen, Qiong Wang, Jiajia Fu, Qianqian Wei, Ruwei Ou, Xiaohui Lai, Xueping Chen, Huifang Shang

**Affiliations:** 1 Department of Neurology, West China Hospital of Sichuan University, No. 37 Guoxue Road, Chengdu, 610041 Sichuan China; 2 Department of Critical Care Medicine, West China Hospital of Sichuan University, Chengdu, Sichuan China

**Keywords:** Spinal muscular atrophy, Serum biomarkers, Nusinersen, Correlation analysis

## Abstract

**Background:**

The creatinine to cystatin C ratio (CCR) can be used as a biomarker of muscle mass and strength, but no studies have evaluated whether it can be used as a biomarker to monitor the efficacy of treatment with nusinersen in Chinese adults with 5q-associated spinal muscular atrophy (SMA).

**Methods:**

In this prospective observational study, 28 adult SMA patients were followed for 18 months. Data on motor function and daily activities were collected using the Hammersmith Functional Motor Scale Expanded (HFMSE), Revised Upper Limb Module (RULM), 6-Minute Walking Test (6WMT), and Barthel Index (BI). Serum levels of creatinine (Cr), creatine kinase (CK), and CCR were measured. Spearman correlation and linear mixed models analyzed the relationships between functional scores and laboratory indicators.

**Results:**

HFMSE scores showed significant improvement at Visit 5 (V5) [+ 1.04 points, *p* = 0.016), V6 (+ 1.61, *p* = 0.012), V7 (+ 1.93, *p* < 0.001), and V8 (+ 1.89, *p* < 0.001)], while RULM scores improved significantly at V5 (+ 2.00 points, *p* = 0.024), V7 (+ 2.00, *p* = 0.032), and V8 (+ 2.00, *p* < 0.001). The BI also exhibited significant improvement at V7 (+ 5.00, *p* < 0.001) and V8 (+ 2.50, *p* < 0.001). The 6MWT did not show significant improvement (*p* > 0.05). Serum CCR levels were significantly higher than baseline at V5 (+ 4.35 points, *p* < 0.001), V7 (+ 5.12, *p* < 0.001), and V8 (+ 6.59, *p* < 0.001). Cr levels were significantly higher than baseline at V5 (+ 2.39 points, *p* < 0.001) and V7 (+ 0.90, *p* < 0.001), while log10 CK levels remained unchanged (*p* > 0.05). Spearman correlation analysis revealed CCR showed the strongest correlation with functional scores, followed by Cr and log10 CK. Further linear mixed-effect model indicated that after adjusting for covariates, only CCR showed a dynamic positive correlation with HFMSE scores (*β* = 0.280; 95% CI 0.023–0.537, *p* = 0.033), while other serum indicators had no correlation with functional scores.

**Conclusion:**

Long-term use of nusinersen effectively improves motor function and quality of life in adult SMA patients, and CCR may be a better indicator to reflect changes in motor function during treatment compared to other blood biomarkers.

**Supplementary Information:**

The online version contains supplementary material available at 10.1186/s13023-025-03730-3.

## Introduction

Spinal Muscular Atrophy (SMA) is a rare neurodegenerative disorder caused by mutations in the *SMN1* gene, which encodes the survival motor neuron (SMN) protein. The reduced expression of SMN leads to the loss of α-motor neurons, progressive muscle weakness, and muscle atrophy, often resulting in premature death [1]. Since the majority of SMA patients have their disease locus mapped to the same region on chromosome 5q13, it is classified as a distinct clinical entity known as 5q-SMA [2]. Nusinersen, an antisense oligonucleotide therapy, has been demonstrated to be effective in treating 5q-SMA with increasing evidence including real-world [3–8]. Current studies provide preliminary evidence that nusinersen improves motor function; however, assessments have primarily relied on clinical rating scales. The variability among assessors, limited sensitivity to subtle functional changes, and the presence of ceiling or floor effects collectively constrain the utility of these clinical scales. These limitations are particularly evident in clinical decision-making regarding the initiation, adjustment, or discontinuation of treatment, especially in SMA patients with a longer disease duration. In contrast, combining specific may biomarkers offer a more objective and quantifiable means of monitoring motor function changes, holding promise for optimizing individualized treatment strategies [9]. Consequently, the establishment of objective biomarkers is essential to enhance the decision-making process regarding treatment, as well as to monitor disease progression and response to therapy [9, 10]. Furthermore, given that SMA requires lifelong treatment, biomarkers should be easy to obtain quickly, cost-effective, and suitable for repeated evaluations [11]. Based on the above, blood-derived biomarkers are considered a primary focus for further development.

The phosphocreatine/creatine kinase (PCr/CK) system plays a crucial role in bioenergetic processes within metabolically demanding tissues, with over 90% of the body’s total creatine stored in skeletal muscles [12, 13]. Creatinine (Cr) and CK are key metabolites from skeletal muscle metabolism and serve as measurable indicators of this process. Some studies have indicated that serum Cr and CK levels were correlated with motor function and disease severity in SMA patients [9, 14]. However, longitudinal data remain limited to support these findings, and there were no studies which examined whether these indicators have dynamic correlations to functional scores. Notably, Cr and CK levels can be affected by factors such as diet, kidney function, and strenuous exercise, which may limit their reliability as true indicators of muscle status [13, 15]. In contrast, the creatinine-to-cystatin C ratio (CCR) has been used as an objective indicator for assessing muscle mass and strength in patients with conditions such as diabetes, cancer, and transplantation, as it is less influenced by external factors [16–19]. Currently, there is still no research focusing on whether nusinersen treatment could improve patients’ daily living abilities. Furthermore, there were no objective indicators to reflect changes in these abilities during treatment.

We monitored serum CK, Cr, and CCR in a longitudinal study of adult SMA patients and evaluated their associations with functional scores during nusinersen treatment.

## Methods

### Standard protocol approvals, registrations, and patient consents

An 18-month longitudinal study was conducted at the West China Hospital of Sichuan University, spanning from August 2021 to June 2024, to assess the impacts of nusinersen on individuals with 5q-associated spinal muscular atrophy (SMA). Eligible participants were adults (aged 18 years or older) who received a confirmed genetic diagnosis of SMA. Detailed demographic information and clinical variables, such as age, gender, body mass index (BMI), SMA clinical subtype, *SMN2* copy number, the degree of ambulatory capacity, and the forced vital capacity percentage (FVC%) were comprehensively documented. Patients received nusinersen treatment by intrathecal administration according to the prescribing information for up to 18 months. Concurrently, evaluations were performed at every nusinersen administration. The study protocol consisted of 8 times of measurement of functional tests and venous blood sampling. The battery of functional assessments conducted included the Hammersmith Functional Motor Scale Expanded (HFMSE), Revised Upper Limb Module (RULM), 6-Minute Walking Test (6MWT). Barthel Index (BI) was used to evaluate the daily living abilities. Functional assessments were conducted by the same trained evaluator one day before each Nusinersen treatment. The evaluator had completed the standardised training programme for SMA clinical assessment scales and obtained the corresponding certification. All blood samples were non-fasting and were allowed to clot at room temperature in serum separator tubes (SST, 3.5 mL) for 0.5 to 2 h. Subsequently, these serum samples were subjected to analysis for Cr, CK, and cystatin C at the certified in-house laboratory departments of West China Hospital, employing the Jaffe method or analogous enzymatic reactions. The analyses were conducted following the methodology delineated in our prior research endeavors.

The study received ethical approval from the ethics committees of West China Hospital, and written informed consent was obtained from all participating patients (2023 (1960) Approval).

### Statistical analysis

All statistical analyses were conducted using the SPSS Statistics 27.0 (IBM, Chicago, IL, USA) and GraphPad Prism 10.0 (GraphPad Software Inc., San Diego, CA, USA). All results were corrected using the Bonferroni method, with statistical significance defined as a two-tailed p-value < 0.05. Shapiro-Wilk analyses were conducted to test the normality of the variables. Continuous variables that were normally distributed were shown as means and standard deviations (SDs), and continuous variables that were not normally distributed were shown as medians and interquartile ranges (IQRs), whereas categorical variables were shown as numbers and percentages. Serum CK levels were log-transformed to reduce skewness, and all subsequent statistical analyses were based on the log-transformed CK values. Wilcoxon signed-rank test was used to compare laboratory markers and functional scores of SMA patients at baseline and during subsequent follow-ups, including Visit 5 (V5) (6 months), V6 (10 months), V7 (14 months) and V8 (18 months). Spearman correlation analysis was used to assess the relationship between baseline serum biomarkers and patients’ function scores and other clinical variables, as well as the correlation between biomarkers and motor function scores at each subsequent treatment time point. A linear mixed-effects model was used to explore whether serum biomarker levels could be used to reflect the changes of motor function and quality of life in SMA patients during nusinersen treatment.

## Results

### Participants

Twenty-eight adult patients with SMA type 3 (*n* = 27) and type 4 (*n* = 1) were included in the analyses, and the detailed baseline characteristics of these patients were presented in Table 1. The mean age of the patients was 25.29 ± 8.95 years, and 57.14% were male. Sixteen SMA patients (57.14%) were ambulant who were able to walk independently. Before starting therapy with nusinersen (V1), the mean HFMSE score was 34.75 ± 24.78, and the median RULM score was 31.50 [95% Confidence Interval (CI): 14.00-35.75]. The 6MWT distance for 16 patients averaged 300.13 ± 151.15 m. The median BI score was 67.50 (95% CI: 36.25–83.75). FVC% was assessed in 15 patients, with an average of 86.80 ± 15.90%. The median serum CK level was 196.00 u/L [(95% CI: 87.25–358.50, normal range: 18.0-198.0 u/L)], displaying significant skewness. In contrast, the mean log10-transformed CK (log CK) level was 2.34 ± 0.41 u/L (normal range: 2.89–5.29 u/L), which followed a normal distribution. Serum Cr levels averaged 26.18 ± 11.79 µmol/L (normal range: 44-133umol/L), cystatin C levels averaged 0.89 ± 0.11 mg/L (normal range: 0.51-1.09 mg/L), and the mean CCR was 29.40 ± 12.57 umol/mg.

**Table 1:** Baseline characteristics

Variable	Patients with SMA (*n* = 28)
Age (years)	25.29 ± 8.95
Sex (male/female)	16/12
BMI (kg/m^2^)	19.95 ± 3.75
Disease duration (years)	19.46 ± 7.98
SMA type (3/4)	27/1
*SMN2* copy number (3/>3)	11/17
Number = 3	11
Number = 4	16
Number = 5	1
Ambulant (yes/no)	16/12
HFMSE	34.75 ± 24.78
RULM	31.50 (14.00-35.75)
6MWT (m)	16, 300.13 ± 151.15
BI	67.50 (36.25–83.75)
FVC (n, %)	15, 86.80 ± 15.90
CK (u/L)	196.00 (87.25–358.50)
Log10 CK (u/L)	2.34 ± 0.41
Cr (umol/L)	26.18 ± 11.79
Cystatin (mg/L)	0.89 ± 0.11
CCR	29.40 ± 12.57

BMI: Body Mass Index; HFMSE: Hammersmith Functional Motor Scale Expanded (range 0–66);

RULM, Revised Upper Limb Module (range 0–37); 6MWT: Six Minute Walk Test;

BI: Barthel Index (0-100); CK: serum creatine kinase; Cr, serum creatinine; CCR: the creatinine to cystatin ratio

### Evolution and comparison of indicators and functional scores

**Fig. 1:**
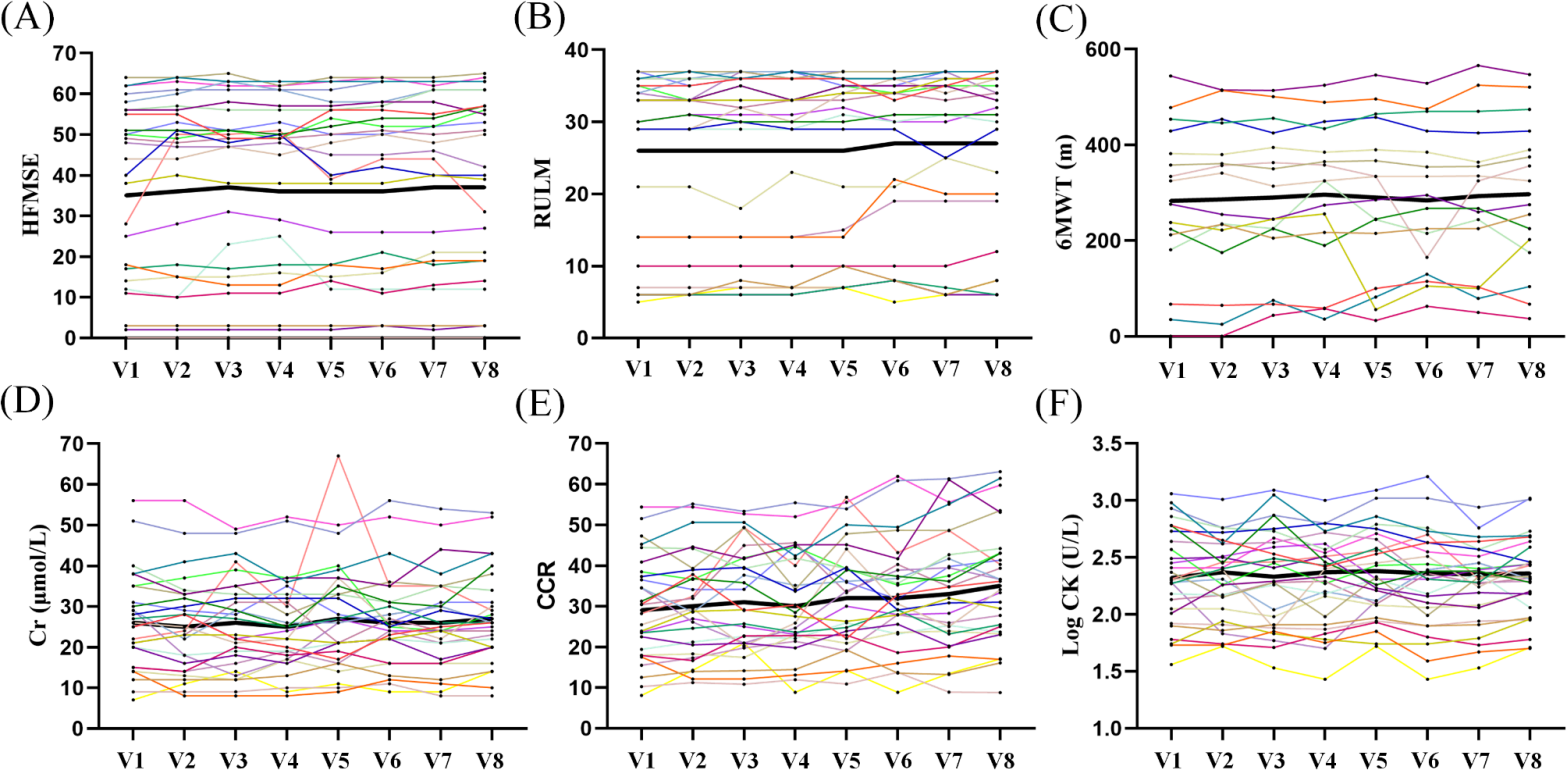
Changes in functional scores and serum biomarkers in SMA patients during nusinersen treatment. The bold black line represents the average change across all patients. HFMSE scores, RULM scores, 6MWT, and CCR exhibited a slight upward trend (**A**-**C**, **E**), while Cr showed a mild increase with fluctuations (**D**). Log CK initially stabilized before displaying a slight downward trend (**F**). HFMSE: Hammersmith Functional Motor Scale Expanded; RULM, Revised Upper Limb Module; 6MWT: Six Minute Walk Test; CK: creatine kinase; Cr, serum creatinine; CCR: the creatinine to cystatin ratio

In this cohort, the functional scores and serum indicators monitored during treatment were shown in Fig. 1. Detailed comparisons of patients’ functional and serum indicators after treatment versus baseline were shown in Table 2. Specifically, HFMSE scores increased significantly from baseline at V5 (mean change + 1.04 points, *p* = 0.016), V6 (+ 1.61, *p* = 0.012), V7 (+ 1.93, *p* < 0.001), and V8 (+ 1.89, *p* < 0.001). RULM scores also showed significant increases from baseline at V5 (median change + 2.00 points, *p* = 0.024), V7 (+ 2.00, *p* = 0.032), and V8 (+ 2.00, *p* < 0.001). Similarly, BI scores significantly improved from baseline at V7 (median change + 5.00, *p* < 0.001) and V8 (+ 2.50, *p* < 0.001). The 6MWT data fluctuated across evaluations post-treatment, reaching the highest value at V8 (median change 310.20 ± 153.45, *p* = 0.332); however, none of the values at any assessment point were statistically significant compared to baseline (all *p* > 0.05). In terms of clinical benefits at V8, 10 patients (35.71%) achieved clinically meaningful improvement with an HFMSE score increase of ≥ 3 points, of whom 8 were ambulant. Eleven patients (39.29%) showed an RULM improvement of ≥ 2 points, with only 3 being ambulant. Twelve patients (42.86%) had an improvement in BI of ≥ 10 points, including 6 ambulant patients. Additionally, 6 patients (37.50%) experienced an increase of more than 20 m in the 6WMT. Cr levels increased significantly at V5 (mean change + 2.39 points, *p* < 0.001) and V7 (+ 0.90, *p* < 0.001), compared to baseline. In terms of laboratory indicators, CCR values also increased significantly at V5 (mean change + 4.35 points, *p* < 0.001), V7 (+ 5.12, *p* < 0.001), and V8 (+ 6.59, *p* < 0.001). However, the log10 CK levels showed a slight downward trend after treatment, but this change was not statistically significant compared to baseline.

**Table 2:** Comparison of serum markers and functional assessments during treatment versus baseline

Variable	Baseline data (V1)	6-month data (V5)	10-month data (V6)	14-month data (V7)	18-month data (V8)
**Log10 CK (u/l)** ^a^	2.34 ± 0.41	2.34 ± 0.37	2.30 ± 0.41	2.28 ± 0.34	2.33 ± 0.34
		*p* = 0.879, *p1* = 3.516	*p* = 0.330, *p1* = 1.320	*p =* 0.053, p1 = 0.212	*p =* 0.733, *p1* = 2.932
**Cr (umol/l)** ^a^	25.50 ± 11.94	27.89 ± 13.35	26.71 ± 11.11	26.46 ± 11.46	27.43 ± 11.37
		***p*** **< 0.001**	*p* = 0.784	***p*** **< 0.001**	*p* = 0.072
**CCR** ^a^	28.63 ± 12.46	32.98 ± 13.15	33.20 ± 12.78	33.75 ± 14.47	35.22 ± 14.24
		***p*** **< 0.001**	*p* = 1.028	***p*** **< 0.001**	***p*** **< 0.001**
**HFMSE** ^a^	34.75 ± 24.78	35.79 ± 22.93	36.36 ± 22.23	36.68 ± 23.21	36.64 ± 23.39
		***p*** **= 0.016**	***p*** **= 0.012**	***p*** **< 0.001**	***p*** **< 0.001**
**RULM** ^b^	31.50 (14.00–36.00)	33.50 (14.25-36.00)	33.5000 (19.50–36.00)	33.50 (19.25-37.00)	33.50 (19.25-37.00)
		***p*** **= 0.024**	*p* = 0.052	***p*** **= 0.032**	***p*** **< 0.001**
**6WMT (m)** ^a^	300.13 ± 151.15	295.07 ± 146.42	307.60 ± 152.78	300.13 ± 151.15	310.20 ± 153.45
		*p* = 3.032	*p* = 3.044	*p* = 2.212	*p* = 0.332
**BI** ^b^	67.50 (36.25–83.75)	70.00 (36.25-85.00)	70.00 (36.25-90.00)	72.50 (36.25-90.00)	70.00 (40.00–95.00)
		*p* = 0.800	*p* = 0.076	***p*** **= 0.008**	***p*** **< 0.001**

HFMSE: Hammersmith Functional Motor Scale Expanded; RULM, Revised Upper Limb Module; 6MWT: Six Minute Walk Test; BI: Barthel Index; CK: serum creatine kinase; Cr, serum creatinine; CCR: the creatinine to cystatin ratio; *p*: P-value obtained after Bonferroni correction. Font bold: *p* < 0.05

^a^ Paired-sample T-test;

^b^ Wilcoxon’s signed rank test

**Table 3:** Correlations between CK, Cr, CCR levels and basial functional assessment

variable	Log10(CK)	CCR	Cr
	(ρ, *p*-value)		
Age (year)	-0.108, *p* = 0.584	-0.075, *p* = 0.704	-0.042, *p* = 0.833
Sex (male/female)	-0.335, *p* = 0.081	0.197, *p* = 0.316	0.045, *p* = 0.821
BMI (kg/m^2^)	-0.194, *p* = 0.332	-0.150, *p* = 0.446	-0.225, *p =* 0.250
*SMN2* copy number (3/>3)	0.321, *p* = 0.096	**0.507**, ***p*** **= 0.006**	**0.566**, ***p*** **= 0.002**
Ambulant (yes/no)	**0.478**, ***p*** **= 0.010**	**0.795**, ***p <*** **0.001**	**0.760**, ***p*** **< 0.001**
HFMSE	**-0.532**, ***p*** **= 0.004**	**0.939**, ***p*** **< 0.001**	**0.897**, ***p*** **< 0.001**
RULM	**-0.598**, ***p <*** **0.001**	**0.872**, ***p <*** **0.001**	**0.806**, ***p*** **< 0.001**
6WMT (m)	0.467, *p* = 0.059	**0.823**, ***p*** **< 0.001**	**0.698**, ***p =*** **0.002**
BI	**-0.525**, ***p*** **= 0.004**	**0.839**, ***p*** **< 0.001**	**0.765**, ***p*** **< 0.001**
Log10 CK (u/l)	/	**0.639**, *p* < 0.001	**0.665**, ***p <*** **0.001**
Cr (umol/l)	**0.665**, ***p <*** **0.001**	**0.962**, ***p*** **< 0.001**	**/**
Cystatin (mg/l)	0.108, *p* = 0.586	-0.059, *p* = 0.766	**0.162**, ***p =*** **0.411**
CCR	**0.639**, ***p <*** **0.001**	**/**	**0.962**, ***p <*** **0.001**

BMI: Body Mass Index; HFMSE: Hammersmith Functional Motor Scale Expanded; RULM, Revised Upper Limb Module; 6MWT: Six Minute Walk Test; BI: Barthel Index; CK: serum creatine kinase; Cr, serum creatinine; CCR: the creatinine to cystatin ratio

### Adverse events

No patients discontinued treatment due to lumbar puncture failure. Ultrasound-guided intrathecal injections were used in 6 patients, four of whom had severe scoliosis (two of these patients underwent spinal correction surgery during the treatment period), and two patients were concerned about repeated lumbar punctures. No serious adverse events related to intrathecal nusinersen injections were observed. 7 (25.00%) patients reported mild side effects, primarily low-pressure headaches following the first lumbar puncture. One patient repeatedly complained of severe low-pressure headaches and nausea/vomiting after lumbar punctures, with symptoms gradually improving after V6.

### Correlations between indicators and functional assessment

The Spearman’s correlation analysis at baseline was detailed in Table 3. CCR was positively correlated with *SMN2* copy number (*ρ* = 0.507, *p* = 0.006), ambulant status (*ρ* = 0.795, *p* < 0.001), HFMSE (*ρ* = 0.939, *p* < 0.001), RULM (ρ = 0.872, *p* < 0.001), 6WMT (*ρ* = 0.823, *p* < 0.001), and BI (*ρ* = 0.839, *p* < 0.001). Cr was also positively correlated with *SMN2* copy number (*ρ* = 0.566, *p* = 0.002), ambulant status (*ρ* = 0.760, *p* < 0.001), HFMSE (*ρ* = 0.897, *p* < 0.001), RULM (*ρ* = 0.806, *p* < 0.001), 6WMT (ρ = 0.698, *p* = 0.002), and BI (*ρ* = 0.765, *p* < 0.001). However, log10 CK was negatively correlated with HFMSE (*ρ* = -0.532, *p* = 0.004), RULM (*ρ* = -0.598, *p* < 0.001), and BI (*ρ* = -0.525, *p* = 0.004), but positively correlated with ambulant status (*ρ* = 0.478, *p* = 0.010) and showed no significant correlation with 6WMT (*ρ* = 0.467, *p* = 0.059).

**Fig. 2:**
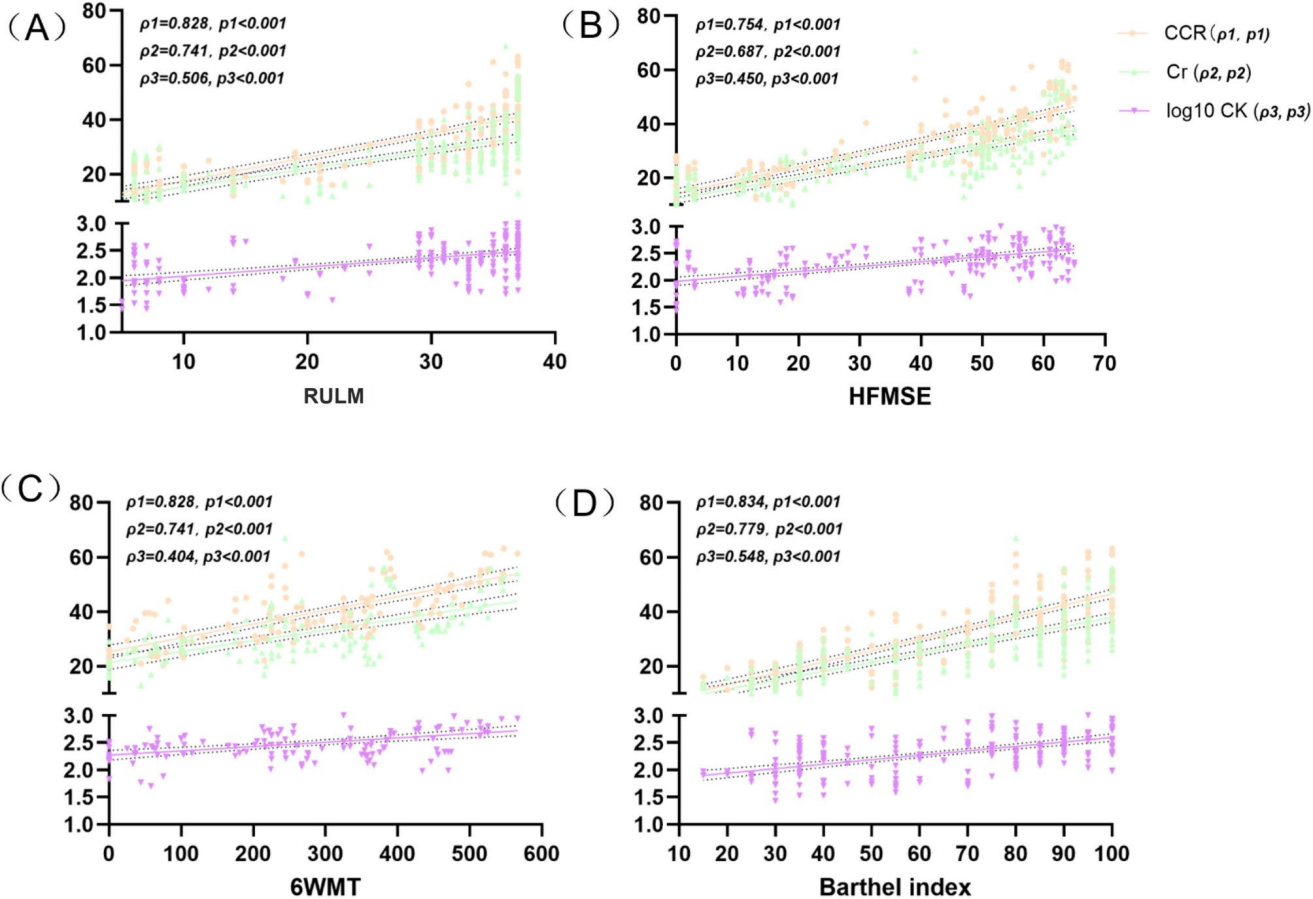
Correlations between the functional data and laboratory parameters during nusinersen treatment. (**A**) Correlation between serum indicators and RULM scores; (**B**) Correlation between serum indicators and HFMSE scores; (**C**) Correlation between serum indicators and 6WMT data; (**D**) Correlation between serum indicators and BI scores. HFMSE: Hammersmith Functional Motor Scale Expanded; RULM, Revised Upper Limb Module; 6MWT: Six Minute Walk Test; BI: Barthel Index; CK: creatine kinase; Cr: creatinine; CCR: the creatinine to cystatin ratio

The dynamic correlations between the functional data and laboratory parameters during treatment were shown in Fig. 2. The correlation coefficients of CCR with HFMSE, RULM, 6WMT, and BI were 0.754, 0.828, 0.828, and 0.834, respectively, indicating that CCR has the highest correlation with functional status (Fig. 2A-D). Cr showed correlation coefficients of 0.687 with HFMSE, 0.741 with RULM, 0.741 with 6WMT, and 0.779 with BI (Fig. 2A-D). In contrast, log10 CK has slight correlations with functional status, with coefficients of 0.450 with HFMSE, 0.506 with RULM, 0.404 with 6WMT, and 0.548 with BI, indicating the lowest correlation with functional assessment data (Fig. 2A-D).

The further adjusted linear mixed-effects model, after correcting for sex, time of treatment, *SMN2* copy numbers, and ambulant status, revealed that only CCR levels were significantly positively correlated with HFMSE scores (*β* = 0.280; 95% CI 0.023–0.537, *p* = 0.033, Table 4).

**Table 4:** Correlation between biomarkers and functional scores in SMA over time

	Variables	Adjusted model	*p*-value
		*β* (95% CI)	
CCR	HFMSE	0.280 (0.023–0.537)	0.033*
	RULM	0.055 (-0.063-0.173)	0.356
	BI	0.290 (-0.476-1.057)	0.455
Cr	HFMSE	-0.120 (-0.391-0.152)	0.635
	RULM	0.031 (-0.097-0.159)	0.635
	BI	0.088 (-0.718-0.894)	0.216
Log 10 CK	HFMSE	0.643 (-3.133-4.418)	0.736
	RULM	-0.283 (-2.166-1.600)	0.766
	BI	-1.425 (-12.324-9.474)	0.796

HFMSE: Hammersmith Functional Motor Scale Expanded; RULM: Revised Upper Limb Module;

6MWT: Six Minute Walk Test; BI: Barthel Index; CK: serum creatine kinase; Cr, serum creatinine;

CCR: the creatinine to cystatin ratio;

*Significant based on the linear mixed-effects model with sex, time, *SMN2* copy numbers and ambulant as covariates;

Font bold: *p* < 0.05

### Subgroup analysis

For non-ambulant patients, the RULM score at V8, CCR at V5 and V8 was significantly higher than baseline, while other functional scores and serum indicators showed no notable improvement (Table S1). For ambulant patients, HFMSE scores showed statistically significant improvements at V6, V7, and V8 compared to baseline, while BI scores reached statistical significance at V7 and V8. RULM scores did not show any significant changes from baseline, and only CCR was significantly elevated at V5, V7, and V8 compared to baseline in ambulant patients (Table S2).

For patients with 3 copies of *SMN2*, significant improvements in RULM scores and CCR were observed at V8 compared to baseline, while other functional scores and serum indicators did not show significant changes (Table S3). For patients with more than 3 copies of *SMN2*, significant improvements in HFMSE and BI scores were observed from V5 to V8 compared to baseline. RULM scores showed significant improvement at V7 and V8. Cr levels reached statistical significance at V8, while CCR was significantly elevated at V5, V7, and V8 compared to baseline (Table S4).

## Discussion

In this prospective study, we identified two key findings: (1) Long-term treatment with nusinersen effectively improved motor function in adult SMA patients across all subtypes and enhanced daily living abilities in patients with *SMN2* copy number > 3 and ambulant. (2) Serum CCR, Cr, and log10 CK levels were correlated with motor function and daily living abilities in adult SMA patients, and CCR was shown to be intensely correlated with functional scores. Further analysis using an adjusted linear mixed-effect model revealed that only CCR was positively associated with changes in HFMSE scores during nusinersen treatment. These results suggested that serum CCR could serve as a potential biomarker for monitoring changes in motor function and daily living abilities in adult SMA patients during treatment.

Our findings are consistent with several real-world reports that have demonstrated the efficacy and safety of nusinersen in treating adult SMA patients [8, 20–22]. However, unlike previous studies, we did not find any statistically significant changes in the 6MWT after treatment compared to baseline, despite a slight upward trend. This may be explained by our relatively small sample size, as well as the variability in patient motivation and external conditions [23]. In our subgroup analysis, we found that the RULM scores in the ambulant group showed an upward trend, but no significant improvement was observed compared to baseline. In the non-ambulant subgroup, we did observe a significant improvement in the RULM scores, with a substantial improvement. However, the non-ambulant group did not exhibit significant improvements in HFMSE scores at any post-treatment assessment, while significant increases in HFMSE and RULM scores were observed in ambulant patents. These findings are consistent with a previous study, which suggested that ceiling and floor effects in the scoring system might explain these results [21]. Notably, our study revealed that adult SMA patients with different *SMN2* copy numbers respond differently to treatment. Patients with 3 copies of *SMN2* experienced more severe symptoms and showed improvement only in RULM scores after treatment. In contrast, patients with more than 3 copies exhibited significant improvements in both RULM and HFMSE scores. These variations in therapeutic efficacy can be attributed to the pharmacological mechanism of nusinersen, with *SMN2* copy number being a crucial factor influencing patients’ responses to the treatment [24].

Our study is the first to use the BI to explore the impact of nusinersen treatment on the daily living abilities in SMA patients. BI is a reliable instrument to evaluate the daily living abilities, since a previous clinical trial also used it to assess the efficacy of valproic acid in SMA, and an improvement of 2.7 points in BI was observed after one year of treatment (*p* = 0.045) [25]. In our study, patients showed the greatest improvement at V7, with a median increase of 5 points. Subgroup analysis revealed that patients with more than 3 *SMN2* copies and ambulant showed significant improvements in BI scores compared to baseline. This finding suggests that the BI score effectively reflects changes in daily living abilities for these subgroups. However, it is noteworthy that in the non-ambulant subgroup, we did not observe a significant improvement in BI scores. The previous study (NCT03032172) found that the spinal muscular atrophy independence scales (SMAIS) only can reflect changes in the daily living abilities of non-ambulant SMA patients during treatment [26, 27]. In the future, combining the BI score with the SMAIS scale may provide a more comprehensive assessment of changes in daily living abilities for adult SMA patients with different subtype.

Although the change in log CK did not reach statistical significance after treatment, it exhibited a mild downward trend. In our cohort, 14 SMA patients (50%) showed significantly elevated serum CK levels before the treatment, consistent with the previous findings [9]. Given that CK is primarily localized in skeletal muscle, its elevation likely results from muscle fiber damage or rhabdomyolysis due to muscle atrophy, leading to increased enzyme leakage [9, 28]. Additionally, increased workload on the remaining fragile muscles may contribute to secondary myopathic changes, further elevating CK levels [13]. In the present study, we applied the log CK as a surrogate for analysis, because serum CK levels did not follow a normal distribution. Previous studies have suggested that long-term nusinersen treatment may help stabilize motor units and improve muscle integrity, including reducing the leakage of intracellular metabolic components such as CK [29–31]. The findings of the present study demonstrate a decline in serum CK levels subsequent to treatment with nusinersen. Following logarithmic transformation, the decline was attenuated, with log CK demonstrating a comparable mild downward trend.

As an increasing number of disease-modifying treatments are being explored for the management of SMA patients, and relying solely on subjective assessments to make decisions about initiating, changing, or discontinuing treatment poses significant challenges, especially for patients with a longer disease duration [9]. In our study, serum Cr was positively correlated with baseline motor function in SMA patients. After 18 months of treatment, while the HFMSE and RULM scores continued to improve from baseline, serum Cr levels did not show significant changes compared to baseline (V8 vs. V1, *p* = 0.072). In contrast, serum CCR at 18 months was significantly elevated from baseline levels, indicating that CCR demonstrates better consistency with dynamic changes in functional scores than Cr. A previous retrospective study found a significant positive correlation between serum Cr and motor function at baseline in SMA patients [9]. However, after 18 months of treatment, serum Cr levels increased only in the non-ambulant group, while levels in the ambulant group decreased, this inconsistence did not correspond with the notable rise in HFMSE scores observed in all patients at 18 months, indicating that serum Cr may not be adequately reflect dynamic changes in motor function [9]. To further investigate, we employed a linear mixed model to assess the correlation between CCR and functional scores over time, accounting for *SMN2* copy number and the patient’s ambulatory status. CCR remained positively correlated with HFMSE scores, supporting the use of CCR as an objective measure for evaluating functional changes in adult SMA patients during nusinersen treatment. CCR reflects muscle mass, which is closely associated with motor function in SMA patients. Therefore, CCR can serve as an indicator for monitoring changes in motor function. Additionally, since Cr is primarily produced by muscle cells and cystatin C is produced by all nucleated cells, CCR is less influenced by renal function and other external factors, making it a more reliable and specific marker than Cr [32]. Numerous studies have also confirmed that the CCR is an effective surrogate biomarker for muscle mass in other patients [16–18, 33]. Although additional research is required to further validate our findings, the use of CCR for long-term monitoring of functional changes in adult SMA patients appears highly promising. This is due to the ease of access to Cr and cystatin C in laboratory tests at hospitals of all levels, along with the straightforward calculation of CCR.

### Limitations

This study has some limitations; this study had a small sample size, was from a single center, and had a relatively short follow-up period. In addition, this study only compared CCR, Cr, and log CK and did not analyze other biomarkers. Future multicenter cohort studies with larger sample sizes and combining multiple metrics are still needed to validate and optimize our findings.

## Conclusion

Nusinersen can effectively improve the motor function and activities of daily living in adult SMA patients, and serum CCR can be used to dynamically monitor the functional changes during the treatment.

## Electronic supplementary material

Below is the link to the electronic supplementary material.

Supplementary Material 1

## Data Availability

The datasets used and/or analyzed during the current study are available from the corresponding author on reasonable request.

## Abbreviations

BI: Barthel Index
Cr: Creatinine
CK: Creatine kinase
CCR: Creatinine to cystatin C ratio
FVC%: Forced vital capacity percentage
HFMSE: Hammersmith Functional Motor Scale Expanded
IQRs: Interquartile ranges
RULM: Revised Upper Limb Module
SDs: Standard deviations
SMA: Spinal muscular atrophy
PCr/CK: Phosphocreatine/creatine kinase
6WMT: 6-Minute Walking Test
