# Supplementary material for: Serum creatinine to cystatin C ratio as monitoring biomarker in Chinese adult spinal muscular atrophy: a prospective cohort study
Source: Orphanet J Rare Dis. 2025 May 2;20:209. doi: 10.1186/s13023-025-03730-3 (PMC12046850; doi:10.1186/s13023-025-03730-3)
Supplement: Supplementary file 1 — Supplementary Material 1 [file 13023_2025_3730_MOESM1_ESM.docx]

Table S1. Changes of variables during treatment versus baseline in no-ambulant subgroup

| **Variable** | **Baseline data**  **(V1)** | **6-month data**  **(V5)** | **10-month data**  **(V6)** | **14-month data**  **(V7)** | **18-month data**  **(V8)** |
| --- | --- | --- | --- | --- | --- |
| **Log10 CK (u/l)** ^a^ | 2.04±0.36 | 2.07±0.29 | 1.94±0.29 | 1.99±0.29 | 2.02±0.24 |
|  |  | *p*=1.988 | *p*=0.140 | *p=*1.160 | *p=*2.932 |
| **Cr (umol/l)** ^a^ | 26.36±15.01 | 27.45±12.94 | 27.27±14.50 | 26.27±15.76 | 26.73±14.13 |
|  |  | *p=*1.584 | *p=*1.132 | *p=*0.296 | *p*=2.580 |
| **CCR** ^a^ | 27.39±14.09 | 32.21±14.43 | 33.21±11.85 | 29.55±16.30 | 32.28±16.64 |
|  |  | ***p=***0.120 | *p=*1.028 | *p* =0.524 | ***p=*0.024** |
| **HFMSE** ^a^ | 11.18±12.21 | 11.64±12.38 | 11.45±12.27 | 12.36±13.12 | 12.55±12.96 |
|  |  | *p*=0.552 | *p*=1.108 | *p=*0.360 | *p=*0.232 |
| **RLUM** ^a^ | 15.91±10.61 | 17.09±10.66 | 17.73±10.50 | 17.73±10.53 | 18.18±11.03 |
|  |  | ***p=*0.044** | *p*=0.152 | *p*=0.116 | ***p=*0.016** |
| **BI** ^b^ | 35.00 (25.00-45.00) | 35.00 (30.00-40.00) | 35.00 (30.00-40.00) | 35.00 (30.00-55.00) | 40.00 (35.00-55.00) |
|  |  | *p=*1.328 | *p*=0.232 | *p*=0.232 | *p=*0.076 |

HFMSE: Hammersmith Functional Motor Scale Expanded; RULM, Revised Upper Limb Module; BI: Barthel Index; CK: serum creatine kinase; Cr, serum creatinine; CCR: the creatinine to cystatin ratio; *p*: P-value obtained from direct comparison; *p1*: P-value obtained after Bonferroni correction. Font bold: *p*< 0.05.

^a^ Paired-sample T-test;

^b^ Wilcoxon's signed rank test.

Table S2. Changes of variables during treatment versus baseline in ambulant subgroup

| **Variable** | **Baseline data**  **(V1)** | **6-month data**  **(V5)** | **10-month data**  **(V6)** | **14-month data**  **(V7)** | **18-month data**  **(V8)** |
| --- | --- | --- | --- | --- | --- |
| **Log10 CK (u/l)** ^a^ | 2.53±0.32 | 2.52±0.31 | 2.30±0.41 | 2.54±0.30 | 2.53±0.24 |
|  |  | *p*=3.472 | *p*=3.284 | *p=*0.476 | *p=*3.468 |
| **Cr (umol/l)** ^a^ | 24.94±9.94 | 28.18±14.00 | 26.35±8.75 | 33.06±9.12 | 27.88±9.64 |
|  |  | *p=*1.064 | *p=*1.464 | *p=*0.064 | *p*=0.068 |
| **CCR** ^a^ | 29.43±11.67 | 33.48±12.69 | 33.20±13.77 | 36.46±12.94 | 37.13±12.63 |
|  |  | *p=*0.032 | *p=*1.632 | ***p*<0.001** | ***p*<0.001** |
| **HFMSE** ^a^ | 50.00±12.40 | 51.41±11.46 | 52.47±10.68 | 52.41±11.49 | 52.24±12.68 |
|  |  | *p*=0.060 | ***p*=0.028** | ***p=*0.020** | ***p*=0.028** |
| **RLUM** ^b^ | 35.00 (31.50-36.50) | 36.00 (33.50-36.50) | 36.00 (33.50-36.50) | 36.00 (33.50-37.00) | 36.00 (33.50-37.00) |
|  |  | *p=*0.352 | *p*=0.648 | *p*=0.332 | *p=*2.120 |
| **BI** ^b^ | 80.00 (75.00-85.00) | 85.00 (75.00-90.00) | 85.00 (75.00-90.00) | 90.00 (77.50-97.50) | 90.00 (72.50-97.50) |
|  |  | *p*=1.284 | *p*=0.144 | ***p*=0.040** | ***p=*0.032** |

HFMSE: Hammersmith Functional Motor Scale Expanded; RULM, Revised Upper Limb Module; BI: Barthel Index; CK: serum creatine kinase; Cr, serum creatinine; CCR: the creatinine to cystatin ratio; *p*: P-value obtained from direct comparison; *p1*: P-value obtained after Bonferroni correction. Font bold: *p*< 0.05.

^a^ Paired-sample T-test;

^b^ Wilcoxon's signed rank test.

Table S3. Changes of variables during treatment versus baseline in *SMN2* copy number of 3 subgroup

| **Variable** | **Baseline data**  **(V1)** | **6-month data**  **(V5)** | **10-month data**  **(V6)** | **14-month data**  **(V7)** | **18-month data**  **(V8)** |
| --- | --- | --- | --- | --- | --- |
| **Log10 CK (u/l)** ^a^ | 2.19±0.46 | 2.19±0.39 | 2.08±0.41 | 2.09±0.38 | 2.11±0.32 |
|  |  | *p*=2.372 | *p*=0.208 | *p=*0.400 | *p=*1.540 |
| **Cr (umol/l)** ^a^ | 26.82±15.18 | 26.18±12.66 | 26.91±14.79 | 18.82±9.70 | 27.09±13.38 |
|  |  | *p=*2.448 | *p=*3.724 | *p=*0.892 | *p* =3.240 |
| **CCR** ^a^ | 29.43±11.67 | 30.78±13.79 | 36.67±15.18 | 31.48±16.04 | 32.97±15.65 |
|  |  | *p=*0.332 | *p=*1.096 | *p=*0.052 | ***p*<0.001** |
| **HFMSE** ^a^ | 31.64±25.74 | 30.09±23.96 | 30.64±24.41 | 30.18±24.12 | 30.73±24.13 |
|  |  | *p*=2.116 | *p*=2.756 | *p=*2.220 | *p=*2.868 |
| **RLUM** ^b^ | 29.00 (6.00-36.00) | 29.00 (7.00-37.00) | 29.00 (8.00-37.00) | 28.00 (6.00-37.00) | 29.00 (8.00-37.00) |
|  |  | *p*=0.168 | *p*=0.108 | *p*=0.556 | ***p=*0.048** |
| **BI** ^b^ | 40.00 (25.00-60.00) | 35.00 (30.00-50.00) | 40.00 (30.00-55.00) | 40.00 (35.00-65.00) | 40.00 (35.00-60.00) |
|  |  | *p=*1.596 | *p*=2.680 | *p*=0.484 | *p=*0.260 |

HFMSE: Hammersmith Functional Motor Scale Expanded; RULM, Revised Upper Limb Module; BI: Barthel Index; CK: serum creatine kinase; Cr, serum creatinine; CCR: the creatinine to cystatin ratio; *p*: P-value obtained from direct comparison; *p1*: P-value obtained after Bonferroni correction. Font bold: *p*< 0.05.

^a^ Paired-sample T-test;

^b^ Wilcoxon's signed rank test.

Table S4. Changes of variables during treatment versus baseline in *SMN*2 copy number greater than 3 subgroups

| **Variable** | **Baseline data**  **(V1)** | **6-month data**  **(V5)** | **10-month data**  **(V6)** | **14-month data**  **(V7)** | **18-month data**  **(V8)** |
| --- | --- | --- | --- | --- | --- |
| **Log10 CK (u/l)** ^a^ | 2.44±0.35 | 2.44±0.34 | 2.45±0.36 | 2.39±0.26 | 2.46±0.36 |
|  |  | *p*=0.776, *p1*=3.104 | *p*=0.776, *p1*=3.104 | *p=*0.469, *p1*=1.876 | *p=*0.368, *p1*=1.472 |
| **Cr (umol/l)** ^a^ | 24.66±9.73 | 29.00±14.05 | 26.59±8.46 | 31.41±9.82 | 27.65±10.31 |
|  |  | *p=*0.516 | *p=*0.792 | *p=*0.108 | ***p=*0.040** |
| **CCR** ^a^ | 29.02±11.39 | 34.41±12.94 | 30.82±10.71 | 35.22±13.67 | 36.68±13.60 |
|  |  | ***p=*0.040** | *p=*3.424 | ***p*=0.004** | ***p*<0.001** |
| **HFMSE** ^a^ | 36.88±21.51 | 38.18±21.67 | 38.76±21.91 | 39.41±21.72 | 39.41±21.72 |
|  |  | ***p*=0.008** | ***p*=0.008** | ***p=*0.004** | ***p<0.001*** |
| **RLUM** ^b^ | 33.00 (25.00-36.0) | 34.00 (25.50-36.00) | 34.00 (26.00-36.00) | 34.00 (27.50-37.00) | 34.00 (26.50-36.50) |
|  |  | *p*=0.184 | *p*=0.816 | ***p*=0.044** | ***p=0.028*** |
| **BI** ^b^ | 80.00 (42.50-85.00) | 85.00 (60.00-90.00) | 85.00 (60.00-95.00) | 85.00 (65.00-95.00) | 90.00 (70.00-97.00) |
|  |  | ***p*=0.032** | ***p*=0.044** | ***p*=0.024** | ***p=*0.032** |

HFMSE: Hammersmith Functional Motor Scale Expanded; RULM, Revised Upper Limb Module; BI: Barthel Index; CK: serum creatine kinase; Cr, serum creatinine; CCR: the creatinine to cystatin ratio; *p*: P-value obtained from direct comparison; *p1*: P-value obtained after Bonferroni correction. Font bold: *p*< 0.05.

^a^ Paired-sample T-test;

^b^ Wilcoxon's signed rank test.
